# Graphene Oxide-Based Biosensors for Liquid Biopsies in Cancer Diagnosis

**DOI:** 10.3390/nano9121725

**Published:** 2019-12-03

**Authors:** Shiue-Luen Chen, Chong-You Chen, Jason Chia-Hsun Hsieh, Zih-Yu Yu, Sheng-Jen Cheng, Kuan Yu Hsieh, Jia-Wei Yang, Priyank V Kumar, Shien-Fong Lin, Guan-Yu Chen

**Affiliations:** 1 Department of Electrical and Computer Engineering, National Chiao Tung University, Hsinchu 300, Taiwan; g199500@gmail.com (S.-L.C.); ericchen161206@gmail.com (C.-Y.C.); viviyou0228@gmail.com (Z.-Y.Y.); shengjen@nctu.edu.tw (S.-J.C.); Kuan.Yu@ibm.com (K.Y.H.); jiawei@nctu.edu.tw (J.-W.Y.); linsf5402@nctu.edu.tw (S.-F.L.); 2 Institute of Biomedical Engineering, College of Electrical and Computer Engineering, National Chiao Tung University, Hsinchu 300, Taiwan; 3 Division of Haematology/Oncology, Department of Internal Medicine, Chang Gung Memorial Hospital (Linkou), Taoyuan 333, Taiwan; wisdom5000@gmail.com; 4 School of Chemical Engineering, University of New South Wales, Sydney, NSW 2052, Australia; priyank.kumar@unsw.edu.au; 5 Department of Biological Science and Technology, National Chiao Tung University, Hsinchu 300, Taiwan

**Keywords:** liquid biopsy, circulating tumor cells, circulating tumor DNA, exosome, graphene oxide

## Abstract

Liquid biopsies use blood or urine as test samples, which are able to be continuously collected in a non-invasive manner. The analysis of cancer-related biomarkers such as circulating tumor cells (CTCs), circulating tumor DNA (ctDNA), microRNA, and exosomes provides important information in early cancer diagnosis, tumor metastasis detection, and postoperative recurrence monitoring assist with clinical diagnosis. However, low concentrations of some tumor markers, such as CTCs, ctDNA, and microRNA, in the blood limit its applications in clinical detection and analysis. Nanomaterials based on graphene oxide have good physicochemical properties and are now widely used in biomedical detection technologies. These materials have properties including good hydrophilicity, mechanical flexibility, electrical conductivity, biocompatibility, and optical performance. Moreover, utilizing graphene oxide as a biosensor interface has effectively improved the sensitivity and specificity of biosensors for cancer detection. In this review, we discuss various cancer detection technologies regarding graphene oxide and discuss the prospects and challenges of this technology.

## 1. Cancer and Diagnosis

Cancer is caused by abnormal cell proliferation and further forms of tumors. Cancer cells are mostly derived from functional changes in genetic materials in the normal cell caused by oncogenic factors and, in a few cases, are caused by parental inheritance of abnormal genes. Activation of oncogene or loss of function of tumor suppressor genes can lead to uncontrolled cell division rates [1]; continued cell growth and division leads to tumor formation [2], which can threaten life by causing organ failure or metastasizing to other sites [3]. Cancer is first diagnosed from medical imaging technologies, such as X-ray, computed tomography, and magnetic resonance imaging. In most cases, physicians will further employ a tissue biopsy to evaluate clinical TNM staging (Classification of Malignant Tumors, T: the size of the primary tumor, N: nearby lymph nodes, M: distant metastasis), depending on the degree of tumor development, lymph node involvement, and distant metastasis [4]. However, traditional detection techniques can often only detect advanced cancers, and general tissue section analysis cannot present complete information about cancer due to the heterogeneity of tumor tissue [5,6]. Yet, physicians require more information to assess treatment strategies, and the detection of specific genes can effectively determine the degree of damage to oncogenes and estimate the future type, and possible target, of metastasis of tumors. Therefore, effective detection of cancer-related biomarkers is an important objective for the clinical diagnosis of cancer. In addition, early diagnosis of cancer and immediate treatment can significantly reduce patient mortality and improve the recovery rate [7].

## 2. Liquid Biopsies

Liquid biopsies have recently emerged as a method for the early detection of cancer [8,9]. It has the advantages of being non-invasive and having a short detection time. Compared to traditional tissue sectioning, which takes several weeks, liquid biopsies require a shorter time, and the collection of only 7–10 mL of blood can screen whether cancer markers are present in the blood. Liquid biopsies primarily screen the blood; because blood circulates through the whole body, the information that can be provided by it is far more clinically significant than results from sections that represent only a certain location [10]. This novel technology is a valuable tool in clinical diagnosis and treatment [11]. Moreover, it also plays an important role in monitoring prognosis, which can be used for regular follow-up [12], and can be processed in a timely manner after recurrence. In addition, it also greatly reduces the discomfort and surgical risk in patients due to repeated sampling. This method can also be used to assess the appropriate dose of medication to a patient [13]. However, current technologies are not yet mature and the sensitivity of detection may also miss these rare but important tumor markers in the blood. Therefore, improving detection sensitivity and developing high-throughput detection are urgently needed to overcome the shortcomings of liquid biopsies [14]. 

## 3. Circulating Tumor Cells (CTCs)

The concept of a liquid biopsy is derived from circulating tumor cells (CTCs), which, as the name suggests, are tumor cells that circulate in the blood. Due to intrinsic or extrinsic factors, the cancer cells of the primary tumor will penetrate the tissue’s basement membrane, enter the bloodstream, and then circulate throughout the body via the blood, thus resulting in metastasis [13]. By monitoring and tracking these cells, it is possible to monitor the course of disease in the patient at any time and adjust the direction of treatment in a timely manner. CTCs play an important role in cancer research and treatment; they originate from a researcher’s “seed and soil” theory published in 1889 [13], where the seeds represent cancer cells and the soil represents the microenvironment preferred by the cancer cells for growth. When the cancer begins to metastasize, it releases many signaling factors similar to fertilizer, which travel through the bloodstream to specific tissues and organs, and then these factors will attract tumors to settle and grow. This phenomenon also explains why cancer metastasis does not occur randomly. The most essential mechanism during the process of cancer metastasis is epithelial–mesenchymal transition (EMT) [15,16], which describes the transformation of the phenotype of some cells in the tumor from densely connected epithelial tissue to more flexible and invasive interstitial cells. This causes normal cells to loosen and provides an opportunity for tumor cells to invade the blood vessels and circulate to the whole body through the blood. When cancer cells reach a suitable environment for growth, they undergo mesenchymal–epithelial transition (MET), the mechanism that is the reverse of EMT, in which cells revert to the original epithelial cells. However, this mechanism of EMT does not represent an absolute negative, it is also an indispensable mechanism for embryogenesis and organogenesis [17].

Due to the extremely low content of CTCs in the blood, a high purity, highly sensitive purification method is required to separate CTCs from a blood sample for detection and subsequent analysis. The current sorting and purification methods can be divided into biochemical separation methods and physical separation methods [18,19]. Biochemical separation methods primarily utilize capturing specific protein markers expressed on the surface of CTCs by antibodies, to separate CTCs from the blood [19]. Physical methods are based on the differences in size and density between CTCs and other blood cells or small molecules in the blood by mechanical or electrical methods for separation, including density centrifugation, filtration, and microfluidic chips [20]. Currently, both purification methods have been applied in clinical experiments, but there are still many issues that must be overcome, especially the loss during purification methods due to the heterogeneity of CTCs. However, combining the two will be a future trend in the development of CTC purification methods [20].

## 4. Circulating Tumor DNA (ctDNA)

The unbalanced cell cycle of cancer cells causes cancer cells to excessively proliferate and die. When the rate of debris clearance of macrophage is lower than the rate of tumor cell death, the content of cell-free DNA (cfDNA) will increase greatly and continue to circulate in the blood [21]. Many studies have shown that in the development of cancer, the concentration of cfDNA in the blood of cancer patients is much higher than in normal people [22]. In 1989, Stroun and colleagues indicated that a small proportion of cfDNA in the serum of cancer patients is released by dying cancer cells. The cfDNA containing cancer genes are also known as circulating tumor DNA (ctDNA) [23]. In recent years, analyzing mutation information in ctDNA from the blood of patients has been used for early diagnosis, formulating follow-up treatment strategy, and assessing prognosis, and it can also be used to monitor treatment efficacy, analyze drug resistance, and discover possible metastasis targets at early stages [24].

The methods currently used to analyze ctDNA can be broadly divided into two categories. The first category is Polymerase chain reaction (PCR)-based techniques [24,25,26,27], which are commonly used to detect single or multiple hotspot mutations in a single gene [24,26,27,28]. One of the most common techniques is droplet digital PCR (ddPCR) [27,29,30,31], in which a sample is separated into multiple independent droplets through different sample isolation methods, such as emulsified droplet or micro-channel techniques [32,33]; each droplet undergoes PCR, then statistical analysis is performed to quantify fluorescent signals in each droplet for absolute quantitation of the target sequence. This method can effectively improve the ctDNA sensitivity of conventional PCR technology and the difficulty in quantitation, and it is currently the primary method for monitoring clinical ctDNA content [24]. The second category of methods uses various next-generation sequencing (NGS) techniques for whole genome sequencing [34,35] and deep sequencing of target regions [33,36,37,38]. These techniques are commonly used to confirm genovariation in patients, to detect potential chromosomal mutations, to evaluate the efficacy of medication and treatment from genotypes, to predict cancer development trends, and to provide timely treatment. However, these ctDNA analysis techniques usually require PCR for sample amplification—this increases background cfDNA concentration and dilutes the detection target, which affects the sensitivity and quantitative evaluation of the assay [30]. In other words, ultra-low concentration ctDNA used in early cancer detection needs a high-sensitivity, high-specificity probe detection platform.

## 5. Exosome

In 1980, Johnstone et al. found that certain transferrin receptors and membrane-associated substances in the reticulocytes of adult mammals selectively release multi-vesicular bodies into the circulatory system, and named them exosomes [39,40,41]. Exosomes are extracellular vesicles released by cells that range in size from 30 to 200 nm [42,43,44] and serve as a medium for intercellular communication [45,46,47]. They are primarily responsible for transporting biologically active molecules such as nucleic acids, proteins, lipids, RNAs (mRNA, miRNA, long non-coding RNA), and DNAs (mtDNA, ssDNA, and dsDNA) [48,49]. Exosomes originate from cancer cells and establish a tumor microenvironment by immune system suppression, angiogenesis promotion, and EMT induction [50], which gives surface markers of exosomes great potential in early cancer diagnosis, metastasis, and monitoring of cancer treatment.

The main bottleneck in the clinical application of exosomes is the lack of efficient isolation methods. Traditional separation methods include differential ultracentrifugation, density-gradient separation, and affinity [51]. Differential centrifugation is based on the difference in size and buoyancy between exosomes and other extracellular vesicles (EVs), but it requires a long centrifugation time and the recovery and specificity is low [52,53]. Density-gradient centrifugation has a higher recovery and purity than differential centrifugation, but the buoyancy and density of exosomes are similar to those of shed microvesicles (sMVs) and viruses [54]. The above-mentioned methods cannot separate exosomes effectively. As a result, many research groups in the past decade have attempted to apply microfluidics to the separation of exosomes. As the name implies, reducing the fluid motion to the micrometer (μm) level in microfluidics, inertia, and gravity in the fluid mechanics at this small scale is negligible. Instead, the viscosity of the fluid becomes a key factor. The microfluid is also closer to the flow pattern of human body fluids in blood vessels. The experimental procedure is shrunk to the size of a chip, which greatly decreases detection time and increases accuracy, while reducing the need for a sample, revealing prospects for precision medicine [55].

Currently, the isolation of exosomes in microfluidic chips mainly relies on affinity [51], in which an appropriate antibody is selected to capture the surface markers of the exosome. The antibody is modified on the substrate or on the surface of magnetic beads to enhance the interaction between the capture probe and the exosomes by taking advantage of the large surface area. Although this technology is very mature, it often faces the bottlenecks of low exosome recovery efficiency and the need for a large amount of sample volume (or cell number) preparation. If these problems can be effectively solved, then breakthroughs in the development of exosome-based early diagnosis will be possible.

## 6. Biomedical Diagnostic Applications of GO

Nanomaterials are a research trend that has begun to be applied in interdisciplinary studies in recent years, especially in biomedical fields. Nanomaterials such as gold nanoparticles, carbon nanotubes, graphene, and graphene oxide (GO) have been mentioned in the literature as having applications in biological probes, tissue engineering, and cancer treatment studies [56,57,58,59,60,61,62,63,64,65,66]. Of these, graphene and GO are especially popular topics of research in the field of biosensors [67,68,69,70,71]. GO possesses oxygen-rich functional groups on the surface. It is easily oxidized, acidified, and easily forms covalent bonds, so it is quite suitable for chemical modification. Moreover, due to the good physicochemical and hydrophilic properties of GO, there have been studies in recent years that have begun to use GO for probes, biological reagent analysis, and biological imaging [72,73], demonstrating its great potential in the biomedical field. In addition, GO has high surface capacity, water solubility, and biocompatibility due to its rich functional groups, which facilitate protein modification as biological probes. Existing literature has indicated that GO can be used as a probe substrate to effectively capture many small biological molecules, DNA, bacteria, and cells [74,75].

## 7. GO-Nanointerface for CTC Diagnosis

**Figure 1:**
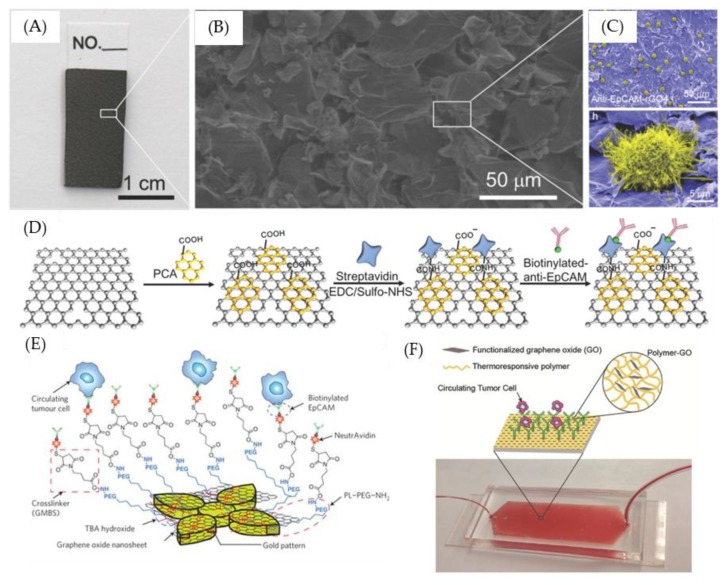
Example of antibody-modified graphene oxide for capturing CTCs. (**A**) A reduce graphene oxide film efficiently captures circulating tumor cells (CTCs) from clinical blood samples. (**B**) Environmental Scanning Electron Microscope (ESEM) image of the reduced graphene oxide (rGO) layer-by-layer structure, and (**C**) an anti- Epithelial cell adhesion molecule (EpCAM) -rGO film after capture CTCs. (**D**) The modification steps of anti-EpCAM-rGO film. (**E**) Schematic of CTC capture system using functionalized graphene oxide (GO) nanosheets on a patterned gold surface. (**F**) Schematic of a polymer–GO microfluidic device. Figures (**A**–**D**) reproduced with permission of [77], Wiley^©^, 2015; (**E**) [76], Springer Nature^©^, 2013; (**F**) [81] Wiley^©^, 2016.

Among the cell capture methods using GO substrates, a popular topic nowadays is the linkage of intact antibodies to GO substrates to capture rare circulating tumor cells or viruses in blood samples [72,76]. Li et al. used a high-temperature method to eliminate the oxygen functional groups on GO to prepare reduced graphene oxide (rGO) in order to capture CTCs with modified antibodies (Figure 1A–D). Because of the rough texture and low stiffness of the rGO surface, the interaction between cells and the biological interface may be enhanced. In addition, because rGO carries a negative charge and is highly hydrophilic, it can prevent non-specific cell adhesion, which effectively improves the capture efficiency of CTCs and reduces background noise. CTCs have been specifically and successfully captured from whole blood containing 10 CTCs/mL [77]. However, there are still some problems with the use of intact antibodies for cell capture that limit their efficiency. For example, the production of intact antibodies is costly, and the production process is also cumbersome. In addition, in order to improve target capture efficiency, additional modification of the GO substrate surface with gold nanoparticles (Figure 1E) [76]. Traditional methods for immobilizing intact antibodies to GO surfaces typically require N-hydroxysuccinimide-lysine(NHS) ester or maleimide chemistries to label free lysine or cysteine residues, respectively, which typically results in intact antibodies immobilized in random orientations and causes lower specificity of the biological probe [72,78]. Using this linkage method also limits subsequent applications; for example, N-hydroxysuccinimide–lysine (NHS) and maleimide–cysteine covalent bonding prevents further linkage of fluorescent groups, which greatly limits fluorescence quantification in the preparation of biological probes. In addition, studies have been conducted to immobilize antibodies to the substrate in a uniform orientation. A common method is to use biotin-streptavidin/NeutrAvidin. However, such methods usually require biotinylation at lysine or cysteine residues. The intermediate proteins required (such as biotin) tend to cause biological probes to have reduced activity [76,79,80], and also causes lower capture efficiency in CTCs. Therefore, related research topics need to not only develop new antibody production technologies to replace intact antibodies but also develop new linkage methods. Another issue in CTC diagnosis is to release the CTCs from the capture nanointerface for subsequent analysis. Yoon et al. developed a thermo-responsive polymer and functionalized GO composite film for the capture/release of CTCs (Figure 1F). They immobilized antibodies to the functionalized GO for capturing CTCs from breast cancer patients’ samples. Then, the captured CTCs could release from the polymer matrix with a lower critical solution temperature (LCST) of 13 °C. The efficient release of captured CTCs from the polymer–GO microfluid make it ideal for various downstream analyses and also shows the potential for liquid biopsies [81].

## 8. GO-Nanointerface for Gene Probe Diagnosis

**Figure 2:**
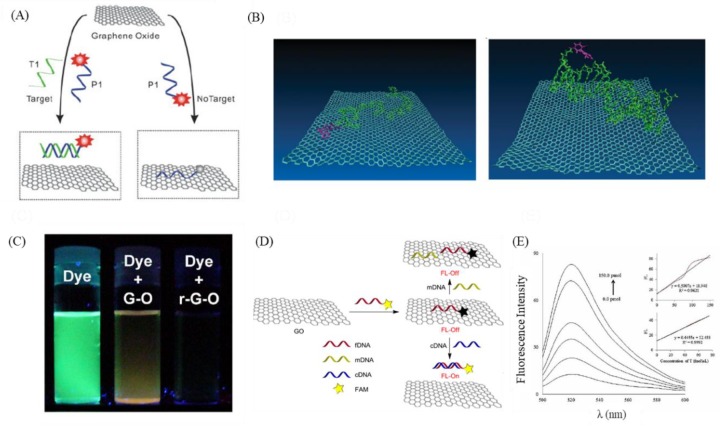
GO-based DNA-based optical sensors. (**A**) Schematic of fluorescent sensors using DNA-functionalized graphene oxide. (**B**) Molecular dynamics simulation of FAM-tagged single—stranded DNA (ssDNA) absorbed on the surface of GO (left) and double—stranded DNA (dsDNA) detached from the surface of GO (right). (**C**) Photographs showing GO and rGO had strong fluorescence quenching ability. (**D**) Schematic of using a DNA-functionalized graphene oxide sensor for deletion mutation in the *EFGR* gene in lung cancer. (**E**) Fluorescence spectra for fDNA after the detection of various concentrations of cDNA. Figures (**A**,**B**) reproduced with permission of [84], Wiley^©^, 2010; (**C**) [100], ACS^©^, 2010; (**D**,**E**) [91], Elsevier^©^, 2016.

Among the literature describing the application of GO in DNA assays, there are many that demonstrate the superiority and sensitivity of GO-based DNA-based sensors that take advantage of the optical, electrical, mechanical, and chemical properties of GO and use the unique features of GO nanostructures and chemical properties [68,82]. These GO sensors can be roughly divided into two types (Table 1). The first type uses GO as a superior receptor for DNA-derived fluorescent probe sensors in Förster resonance energy transfer (FRET) [82,83]. The bases of the DNA probe form a hexagonal honeycomb structure, which interacts with the graphitic (sp2) domains on the GO surface and, through π–π stacking, adsorbs the DNA probe to the GO surface, where the fluorescence is quenched by Förster resonance energy transfer (FRET) (Figure 2A) [69,84,85]. After the DNA probe binds to the target molecule, the binding force between the DNA probe and the target molecule is greater than the binding force between the DNA probe and GO surface, which separates it from the GO surface (Figure 2B). At the same time, a fluorescence recovery signal is generated due to the reduced FRET effect [84]. Many researchers have used this GO as a substrate to develop fluorescent biosensor systems to assay for metal ions, DNA [84,86], RNA [87], small-molecule organic matter [88], peptides, proteins [89], and even cells, and most studies have proven that GO is advantageous for applications related to biological probes. Among them, He et al. used multi-color fluorescent probes to detect specific target sequences and rapidly obtained highly specific and sensitive detection results in complex environmental solutions. They also used this technology to effectively distinguish sequences with single-base errors [84], demonstrating the potential of GO for cancer gene detection. Eftekhari-Sis et al. successfully used this technique to detect exon 19 deletions in the estimated Glomerular filtration rate (*EGFR)* gene, which is a gene mutation that plays a very important role in lung cancer and is used clinically to evaluate the use of targeted drugs in patients with non-small cell lung cancer (Figure 2D) [90]. In addition, it has a linearity of R^2^ = 0.9992 for the detection of the target exon 19 deletion sequence at different concentrations between 0 and 80 fmol/μL, demonstrating that it can detect extremely low concentrations of the target sequence (Figure 2E) [91]. However, there are currently few studies on the application of GO-DNA fluorescent probe optical sensors using FRET for cancer gene detection, because the limits of many such detection techniques fail to detect extremely low concentrations of cancer genes [84]. Thus, improving the limit of detection is a major issue for future research.

**Figure 3:**
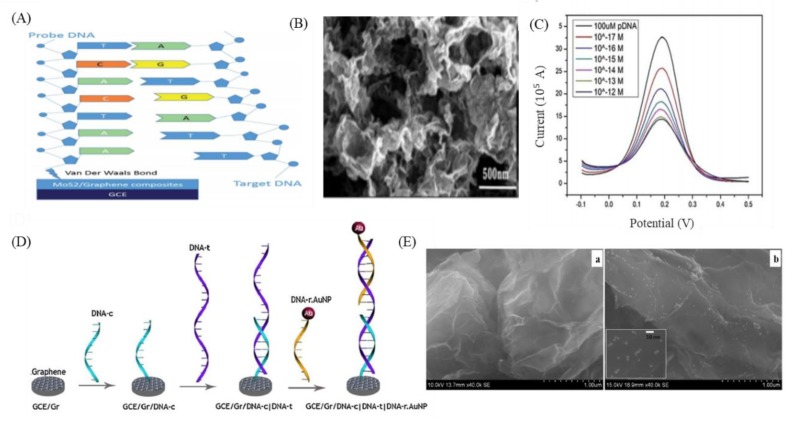
GO-based DNA-based electrochemical sensors. (**A**) Schematic of MoS2/graphene nanosheets electrode for ctDNA detection. (**B**) Scanning Electron Microscope (SEM) image of MoS2/graphene composites. (**C**) The Differential Pulse Voltammetry (DPV) plots change after hybridization of various concentrations of ctDNA. (**D**) Schematic of sensing steps of graphene-DNA electrochemical sensor with AuNPs functionalized report DNA. (**E**) SEM image of sensor without adding DNA-r AuNPs (left) and adding DNA-r AnNPs (right). Figures (**A**–**C**) reproduced with permission of [93], RSC^©^, 2016; (**D**,**E**) [95], Elsevier^©^, 2014.

The second type of GO sensors are electrochemical DNA-based sensors designed to utilize the excellent electrochemical properties of the nanomaterial [67,82,92]. An advantage of electrochemical sensors is that they are label free sensors. By analyzing the electrical signal differences during the interaction between the analyte and the probe, the concentration of the analyte can be derived from analysis of the relevant data [67]. Many studies have indicated that biological detection interfaces using GO as an electrochemical sensor can effectively improve sensitivity. For example, Chu et al. combined MoS_2_ and GO materials using hydrothermal and ultrasonic methods to prepare MoS_2_/graphene nanosheets to improve the electrical conductivity and electrochemical activity of electrochemical DNA-based sensors (Figure 3A,B) [93]. Intermolecular π–π stacking between DNA nucleobases and GO is also beneficial to the sensitivity of single-stranded DNA immobilization on the surface of MoS_2_/graphene nanosheet-modified electrodes, and they can be used to detect ctDNA with a limit of detection of 1 × 10^−17^ M (Figure 3C) [93]. In addition, the combination of gold nanoparticles and GO has been widely used as electrodes for electrochemical sensors [68,94]. Abdul Rasheed et al. used this technique to combine a capture probe (DNA-c) with a reporter probe (DNA-r) to hybridize with target DNA (DNA-t) in a sandwich structure (Figure 3D,E) [95]. Oxidation of the gold nanoparticle modifications of the reporter probe were used for the specific detection of the *BRCA1* gene associated with cancer. The results showed that this sensor had a stable limit of detection of 1 fM [95]. Although there have been many studies reporting that the application of GO to electrochemical sensors can effectively improve sensitivity, the interaction between GO and probes and analytes has not yet been fully elucidated, and the intermolecular forces and electrical properties between them are expected to be confirmed in the future, further enhancing the sensitivity and specificity of this technology and extending its application to ctDNA monitoring and detection at early stages of cancer.

## 9. GO-Nanointerface for Exosome Diagnosis

**Figure 4:**
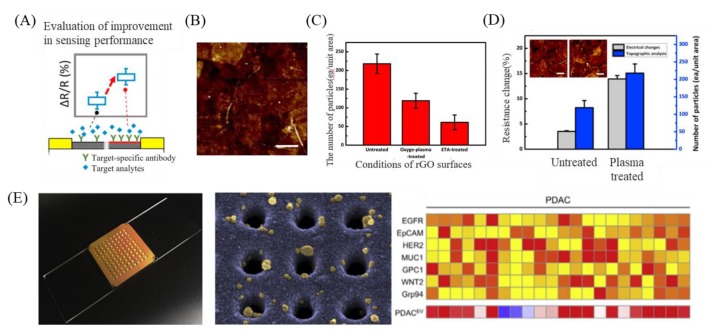
Application of GO-based biosensors for exosome detection. (**A**) Schematic of antibody immobilization on rGO surface. (**B**) Atomic Force Microscope (AFM) image (5 × 5 µm^2^) of antibody-immobilized surface. (Scale bar is 1 µm). (**C**) Efficiency of antibody immobilization on different rGO surfaces. (**D**) Resistance change (Rab-R)/R before and after immobilization and counting the number of immobilized antibodies with a particular size on the AFM image (7–9 nm). (**E**) New nanoplasmonic sensor (NPS) platform and heatmap analysis. Figures (**A**–**D**) reproduced with permission of [102], Elsevier^©^, 2017; (**E**), [104], Elsevier^©^, 2018^.^

Due to GO’s nano-parameter structure and high-compatibility, this material has high potential as an interface of exosome biosensors. Mei Heb et al. modified a GO substrate with a layer of polydopamine (PDA) and used protein G to immobilize antibodies on GO for exosome capture [101]. Chae et al. used oxygen plasma treatment to enhance the reduction of a reduced graphene oxide (rGO) sensor surface for exosome diagnosis in Alzheimer disease patients and found that rGO reduced by oxygen plasma treatment showed a 3.33-fold higher target specificity compared to before treatment (Figure 4A–D). [102]. Wang et al. used DNA aptamers to design a new signal amplification platform for colorectal cancer exosome surface markers CD63 and EpCAM. This method requires only 5 µL of serum sample for the detection of colorectal cancer exosomes. It has significant diagnostic capabilities, confirming that the platform could not only be used for colorectal cancer exosomes, but also for other cancer exosomes [103]. Hyungsoon Im et al. designed a nanoplasmonic (NPS) platform for high-throughput EV analysis. The combination of GO-based interface and heatmap means that EV markers analysis can quickly and sensitively measure 7 biomarkers in 100 samples, as shown in Figure 4E [104]. Cancer-derived circulating exosome play an important role in cancer diagnosis, and moreover, people have tried to use exosomes as an innovative clinical treatment [105]. However, the current exosome detection methods are low recovery or non-specific. The combination of materials and interface modification for exosome detection is indispensable. 

## 10. GO-3D Printing and Micropatterning for Diagnosis

Currently, some research groups are utilizing GO to enhance cancer-related molecule capture by improving the problem of low reproducibility caused by an insufficient sample number in the blood. It can be combined with micropatterning, including soft lithography [106], electron beam lithography [107], microcontact printing [108], self-assembled monolayer [109], and inkjet printing [110], as a novel technique to help capture cancer-related cells in the biosensor system. However, when safety, time, and cost are considered, inkjet printing is simple, fast, inexpensive, and non-contact at the same time, which is conducive to large-scale production [111]. Also, it is applied in low-temperature environments, so changes in the material properties of printing are avoided and printing is possible on a variety of biomaterials. In addition, the inkjet distance, content, and size are automatically calculated by a computer program, which ensures high sensitivity and reproducibility of experiments and makes it suitable for related biochip research [112].

## 11. GO-3D Printing and Micropatterning in CTC, ctDNA, and Exosome Diagnosis

**Figure 5:**
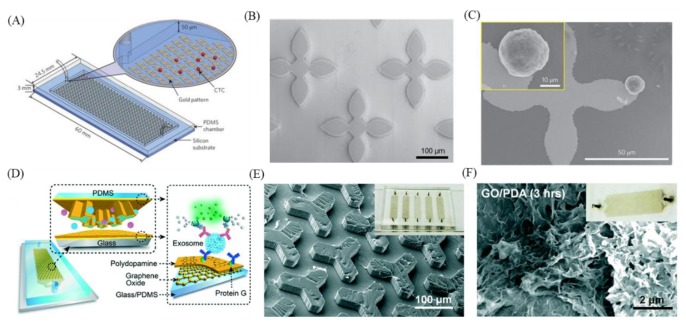
Examples of microfluidic devices with microposts for capturing tumor cells. (**A**) Schematic of circulating tumor cell capturing system with microposts. (**B**) SEM image of flower shaped microposts. (**C**) SEM image of flower shaped micropost with captured tumor cell. (**D**) Schematic of nano-interfaced microfluidic exosome platform and Graphene oxide/polydopamine (GO/PDA) coated interface. (**E**) SEM image of Y shaped microposts with GO/PDA coating. (**F**) SEM image of GO/PDA-coated channel. Figures (**A**–**C**) reproduced with permission of [76], Springer Nature^©^, 2013; and (**D**–**F**) [101], RSC^©^, 2016. This article is licensed under a Creative Commons Attribution-NonCommercial 3.0 Unported Licence.

As mentioned above, many researchers have used micropatterning to increase the capture efficiency of CTC [113,114], DNA [115], and exosomes [116]. For example, in addition to increasing the contact surface area, microposts can be used to generate turbulence to increase the uniformity of sample mixing [117]. There are also some studies that used different printing patterns of GO mixtures calibrated to detect cancer-related markers [118,119]. Yoon et al. used GO modified with EpCAM antibodies deposited on flower-shaped gold microposts on a flat surface to capture CTCs expressing EpCAM that are present in the blood at early stages of cancer (Figure 5A–C) [76]. The results of cell capture tests using human breast cancer cell lines (MCF-7) showed that the micropatterned silicon substrate had an MCF-7 cell recovery rate of 48% and the GO pattern microposts had a minimum MCF-7 recovery rate of 73%, and even reached 100%. In addition, Zhang et al. used a photo-etching technique to create a Y-shaped mold, and then a Polydimethylsiloxane (PDMS) molding technique was used to produce a special nano-interfaced microfluidic exosome platform (nano-IMEX) pattern (Figure 5D–F) [101]. Because of the three-dimensional Y-shaped microposts, it not only allows fluid to periodically mix with the surrounding liquid, and the asymmetric flow caused by the Y-shaped structure allows larger vesicles to flow to the surface then be captured, but it can also increase mixing efficiency, thereby increasing the efficiency of specific immunological exosome capture, while reducing non-specific exosome capture. After modifying the GO on the microposts, polydopamine (PDA) is directly added to the GO, which will self-synthesize and form a nano-structural interface that increases surface area and antibody binding efficiency. Labeled exosomes can be specifically captured using auxiliary CD63, CD81, and EpCAM antibodies. The authors also mentioned that nano-IMEX can be used to directly quantify circulating exosomes in 2 µL of untreated blood. 

**Figure 6:**
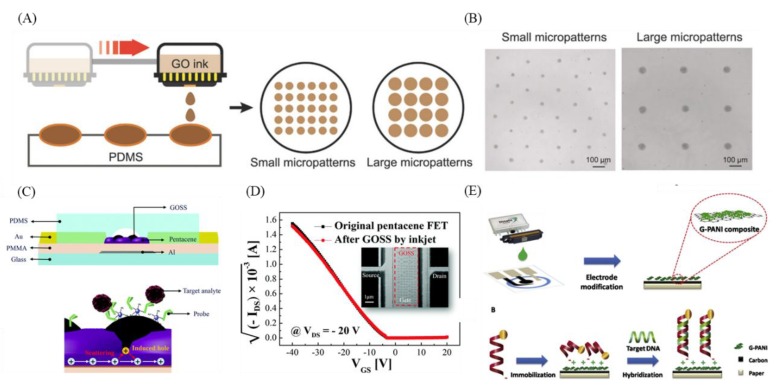
Examples of inkjet printing utilized in tumor-related molecule sensing. (**A**) Schematic of inkjet printing graphene oxide. (**B**) Uniformly inkjet-printed different sizes of graphene oxide micropattern. (**C**) Schematic of graphene oxide support system (GOSS) and its detection mechanism. (**D**) Electrical performance of original pentacene field-effect transistor (FET) and inkjet-printed pentacene FET. (**E**) Schematic of paper-based electrochemical biosensor and its sensing mechanism. Figures (**A**,**B**) reproduced with permission of [127], Wiley^©^, 2018; (**C**,**D**) [120], RSC^©^, 2017; and (**E**) [122], Elsevier^©^, 2017.

In addition, some of the characteristics of inkjet printing, such as its stability and reproducibility, have been applied in a case using GO to detect cancer. Lee et al. used the principle of the field-effect transistor to create a GO support system (GOSS) for pentacene-based field effect transistor (FET)s to detect target DNA and Circulating tumor microemboli (CTM) (Figure 6C,D) [120]. Using the malleable and biocompatible pentacene as the active layer in the FET system, inkjet printing was used to inject materials such as GOSS, which can increase the linkage between antibodies and target DNA, onto the pentacene. Finally, GO was modified with probe DNA or breast cancer-specific antibody (HER2) to capture target DNA and CTM (SkBr3). The electrical analysis will differ when the system is hybridized with DNA or CTM. In addition, the DNA of HPV (human papilloma virus), which is associated with cervical cancer, can be used to determine the presence of cervical cancer [121]. Teengam et al. developed an inexpensive, disposable HPV-DNA detection and monitoring device using inkjet printing with graphene-polyaniline (G-PANI) conductive ink to modify a paper-based electrochemical biosensor (Figure 6E) [122]. The device has advantages for large-scale production, stability, and reproducibility and has reduced demand for samples. In addition, it is modified with a synthetic anthraquinone-labeled pyrrolidinyl peptide nucleic acid (acpcPNA) probe (AQ-PNA) to detect papillomavirus (HPV) type 16 DNA. The current decreases greatly after the target is added, and the degree of capture is determined. In addition to inkjet printing, there have been reports of a device using screen printing of GO for determining blood α-amylase concentration to detect pancreatic and lung cancer [123]; blood α-amylase concentration may be associated with pancreatic [124] and lung cancer [125]. Teixeira et al. developed an α-amylase immunosensor platform by electropolymerization of aniline on a screen-printed graphene electrode, forming a layer of polyaniline film. This film can transfer electrons to the underlying graphene layer, immobilization of α-amylase antibody allows capture of α-amylase molecules, and the α-amylase signal is quantified using the principles of electrochemical impedance spectroscopy (EIS). The signal has a linear response when the concentration of α-amylase is between 1 and 1000 international units/L (IU/L). Similarly, screen printing has been used to print graphene, which was used by Haque et al. for the diagnosis and evaluation of DNA methylation to distinguish between different types of cancer.

With respect to specific epigenetic indicators of the degree of DNA methylation, disease diagnosis and prognosis can be estimated by the degree of methylation, because different cancers have different degrees of methylation in specific body sites [126]. Through the affinity between DNA bases and graphene, the authors obtained and processed single-stranded DNA from cells and added it to the surface of graphene-modified screen-printed carbon electrodes (g-SPCE), along with $Fe{\left(CN\right)}_{6}{}^{\left(3-/4-\right)}$. If there is single-stranded DNA with a high degree of methylation, the DPV current will be lower on conventional differential pulse voltammetry analysis. The authors used the above methods with esophageal squamous cell carcinoma (ESCC) cell lines and esophageal squamous cell carcinoma cells to determine the degree of FAM134B promoter gene methylation and the degree of FAM134B gene expression.

## 12. Outlook

With the advancements in hardware technology and the development of nanomaterials, the combination of GO and biosensors provides great benefits in the detection of clinical biomarkers. Not only does it provide faster and easier detection methods, it also reduces analysis time compared to traditional biological analysis. With advances in various fields, interdisciplinary collaboration is indispensable in order to meet clinical needs, including electrodes, nanomaterials, signal processing, and biomedicine. We expect that the combination of GO and biosensors mentioned in this paper will be developed and further help liquid biopsies; this non-invasive and novel diagnosis method can be used in clinical detection. Moreover, semiconductor materials will no longer be limited to their original form, but will be combined with other biomaterial interfaces to develop fast and convenient detection platforms with high sensitivity and high biocompatibility.

## Figures and Tables

**Table 1:** Performance comparison between GO-based DNA sensors for DNA detection.

Type of GO-Based DNA-Based Sensors	Description of Method	Sensitivity	References
Optical	Multicolor fluorescent DNA nanoprobe	100 pM	[84]
	Fluorescein amidites (FAM) labeled DNA probe	1 nM	[91]
	Molecular beacon	2 nM	[96]
	DNA probe and DNA-intercalating dyes (SYBR Green I)	0.31 nM	[97]
Electrochemical	MoS2/graphene nanosheet-modified electrodes	0.01 fM	[93]
	Gold nanoparticle labeled reporter DNA (DNA-r.AuNP) and DNA-c modified glassy carbon electrode (GCE)/Gr	1 fM	[95]
	PP3CA/ERGO/GCE	3 fM	[98]
	cDNA2 modified AuNPs with catalyzed silver staining and GCE-GR/cDNA1	72 pM	[99]
